# CSF-Targeted Proteomics Indicate Amyloid-Beta Ratios in Patients with Alzheimer's Dementia Spectrum

**DOI:** 10.1155/2023/5336273

**Published:** 2023-02-06

**Authors:** Maryam Behzad, Negin Zirak, Ghazal Hamidi Madani, Linda Baidoo, Ali Rezaei, Shima Karbasi, Mohammad Sadeghi, Mahan Shafie, Mahsa Mayeli, Alzheimer's Disease Neuroimaging Initiative

**Affiliations:** ^1^NeuroTRACT Association, Students' Scientific Research Center, Tehran University of Medical Sciences, Tehran, Iran; ^2^Department of Chemistery, University of Tehran, Iran; ^3^Department of Educational Science and Psychology, University of Tabriz, Tabriz, Iran; ^4^Department of Biology, Faculty of Sciences, University of Guilan, Iran; ^5^Department of Medicine, Isfahan University of Medical Sciences, Isfahan, Iran; ^6^School of Medicine, Tehran University of Medical Sciences, Tehran, Iran; ^7^Alzheimer's Disease Neuroimaging Initiative, USA

## Abstract

**Background:**

According to recent studies, amyloid-*β* (A*β*) isoforms as cerebrospinal fluid (CSF) biomarkers have remarkable predictive value for cognitive decline in the early stages of Alzheimer's disease (AD). Herein, we aimed to investigate the correlations between several targeted proteomics in CSF samples with A*β* ratios and cognitive scores in patients in AD spectrum to search for potential early diagnostic utility.

**Methods:**

A total of 719 participants were found eligible for inclusion. Patients were then categorized into cognitively normal (CN), mild cognitive impairment (MCI), and AD and underwent an assessment of A*β* and proteomics. Clinical Dementia Rating (CDR), Alzheimer's Disease Assessment Scale (ADAS), and Mini Mental State Exam (MMSE) were used for further cognitive assessment. The A*β*42, A*β*42/A*β*40, and A*β*42/38 ratios were considered as means of comparison to identify those peptides corresponding significantly to these established biomarkers and cognitive scores. The diagnostic utility of the IASNTQSR, VAELEDEK, VVSSIEQK, GDSVVYGLR, EPVAGDAVPGPK, and QETLPSK was assessed.

**Results:**

All investigated peptides corresponded significantly to A*β*42 in controls. In those with MCI, VAELEDEK and EPVAGDAVPGPK were significantly correlated with A*β*42 (*p* value < 0.001). Additionally, IASNTQSR, VVSSIEQK, GDSVVYGLR, and QETLPSK were significantly correlated with A*β*42/A*β*40 and A*β*42/38 (*p* value < 0.001) in this group. This group of peptides similarly corresponded to A*β* ratios in those with AD. Eventually, IASNTQSR, VAELEDEK, and VVSSIEQK were significantly associated with CDR, ADAS-11, and ADAS-13, particularly in MCI group.

**Conclusion:**

Our research suggests potential early diagnostic and prognostic utilities for certain peptides extracted from CSF-targeted proteomics research. The ethical approval of ADNI is available at ClinicalTrials.gov with Identifier: NCT00106899.

## 1. Introduction

Alzheimer's disease (AD) is a progressive neurodegenerative disorder with substantial memory loss, executive dysfunction, disorientation, and psychiatric symptoms, enduring 8 to 10 years and affecting individuals and their families [1–3]. AD is the most common cause of dementia [4] and will likely increase to 12.5 million in the United States by 2050 [5]. As a disorder accompanied by cognitive symptoms [6], different clinical cognitive assessments such as the Montreal Cognitive Assessment (MoCA), the Mini-Mental State Examination (MMSE), Mini-Cog, and the Alzheimer's disease Assessment Scale (ADAS-cog) are widely developed and used along with lab assessments to fully picture the disease [7, 8].

Cerebrospinal fluid (CSF) is a proximal fluid characterized by a dynamic range of protein abundance [9]. CSF originates from the ependymal cells in the choroid plexus of the brain's ventricles [10], so CSF proteomics can perfectly mirror the biochemical changes in the central nervous system [11]. A recent CSF proteomics analysis reported that 48 proteins were remarkably dysregulated in AD patients compared to other neurodegenerative diseases [12, 13]. An integrative approach can be useful to advance AD brain network proteomics in identifying promising CSF biomarkers. Several protein targets were previously identified by integrative proteomics linked to brain-based pathophysiology, including dysfunction in synaptic, myelination, and metabolic pathways. Targeted CSF proteomics levels such as APOE, VGF, and CH3L1 altered between AD and control groups and showed consistent changes (up- or downregulation) across independent studies. VGF protein and its related peptides refer to a stimulatory role in memory formation in the hippocampus. They have been implied as AD biomarkers and change with the progression of the disease [14–16]. The increase in secreted protein levels like chitinase 3 like 1 (CHI3L1) and osteopontin (SPP1) was observed in patients with pathology of AD and neurodegenerative disease [17–19]. Also, 14-3-3-Zeta (YWHAZ), which is a signaling protein, showed a high correlation with AD in a recent study (*p* = 3.44 × 10^−3^) [20].

Various biomarkers are used for better risk assessment, diagnosis, prognosis, and monitoring intervention in AD [21]. The three important CSF biomarkers for AD diagnosis are beta-amyloid isoforms, tau protein, and phosphorylated tau protein [11].

The amyloid-beta proteins are formed from the breakdown of a larger protein called amyloid precursor protein. The pathological decline associated with AD is driven by protein, specifically the aggregation of amyloid-beta (A*β*) into neurotic plaques that collect between neurons and disrupt cell function, so there is a noticeable decline in the ratio of A*β*42/A*β*40 in CSF [22], which is greater than the reduction of CSF A*β*42 itself [13]. Research suggests that the abnormal processing of amyloid precursors leads to the development of pathologic plaques [23, 24].

In AD treatment, it is important to reduce the pace of cognitive decline. As amyloid CSF levels can be used as a biomarker for the existence of AD pathology, which can already be identified in the very early grade of the disease when cognition and behavior are still normal [25, 26]. Thus, the importance of hallmark biomarkers that ease the early diagnosis is significant [27] and allows the development of particular tests for the early detection of AD in at-risk groups [28].

Despite many pilot studies, the knowledge about the pathology and heterogeneity of AD is still insufficient because of the absence of duplicate findings. Also, methodological challenges can limit the performance of these biomarkers in clinical practice, especially in the early stages [29, 30]; thus, additional diagnostic tools are beneficial for early and correct diagnosis [31].

The correlation between CSF proteins and cognitive decline should also be investigated to further determine the functionality of these markers in clinical settings.

In this research, we hypothesized that the levels of this CSF proteomics might associate with the rate of cognitive decline in AD patients. Thus, the ability of these CSF biomarkers to predict the rate of disease progression and cognitive function has indications for the early diagnosis and treatment of AD. Therefore, we aimed to investigate the association between the targeted proteomics of CSF samples with the A*β*42/A*β*40 ratio, ADAS-cog-13, and ADAS-total in AD and non-AD groups.

## 2. Methods

### 2.1. Participants

Data used in the preparation of this article were obtained from the Alzheimer's Disease Neuroimaging Initiative (ADNI) database (http://adni.loni.usc.edu). The ADNI was launched in 2003 as a public-private partnership led by Principal Investigator Michael W. Weiner, MD. The primary goal of ADNI has been to test whether serial magnetic resonance imaging (MRI), positron emission tomography (PET), other biological markers, and clinical and neuropsychological assessment can be combined to measure the progression of mild cognitive impairment (MCI) and early Alzheimer's disease (AD). Inclusion and exclusion criteria for ADNI participants are fully described elsewhere. In brief, enrolled subjects aged between 55 and 90 and were required to undergo various test procedures including neuroimaging, lumbar punctures, and longitudinal follow-up. Participants were excluded if they had a Hachinski Ischemic Score of higher than 4, unpermitted medications use, or permitted medications to change over four weeks before the study, a Geriatric Depression Scale score of equal to or higher than 6, less than 6 grades of education or working equivalent. Clinical classification of Alzheimer's disease based on ADNI criteria was used to categorize participants into three cognitively normal (CN), MCI, and AD groups, which is described elsewhere.

We included all subjects with CN, MCI, and AD diagnosis from ADNI database, for whom data regarding CSF-targeted proteomics and CSF amyloid-*β* measurements were available. Besides, we extracted data regarding demographics, diagnosis (CN, MCI, and AD), and cognitive assessments including Clinical Dementia Rating (CDR), Alzheimer's Disease Assessment Scale (ADAS), and Mini Mental State Exam (MMSE), and merged all data tables for further analysis. More specifically, data for CSF-targeted proteomics were extracted from “Emory University CSF targeted MS.csv”; data for CSF amyloid-*β* measurements were extracted from “UPENN–2D UPLC tandem mass spectrometry measurements of Abeta42, Abeta40, and Abeta38”; and data for demographics, diagnosis, and cognitive assessments from “ADNIMERGE–Key ADNI tables merged into one table.csv” from ADNI database. Eventually, a total number of 719 participants were found to be eligible for the study, including 225 CN, 381 MCI, and 113 AD subjects.

### 2.2. Cognitive Assessments

In this study, we used CDR, ADAS, and MMSE to assess cognition, which are valid assessment tools for cognitive and noncognitive behavioral dysfunctions among subjects with the dementia spectrum. CDR is a validated instrument for clinical studies on the AD spectrum, which assesses three domains of cognition (memory, orientation, and judgment/problem solving) and three domains of function (community affairs, home/hobbies, and personal care) using structured interviews. The scores for the six domains (ranging from 0 to 3) tested can be summed (CDR Sum of Boxes or CDR-SB), which was used in this study. The ADAS-Cog is a well-known cognitive assessment with raw scores that range from 0 to 70, where higher scores indicate greater cognitive dysfunction. In addition, due to some limitations in the 11-item version of ADAS-Cog, the 13-item version was recommended which has two complementary items including delayed word recall and a number cancellation or maze task, with a score range between 0 and 85. In this study, we used both ADAS-Cog 11- and 13-item versions. The MMSE is also a well-known brief screening instrument for clinical research on cognitive dysfunction with raw scores that range from 0 to 30, where lower scores indicate greater cognitive dysfunction.

### 2.3. CSF-Targeted Proteomics and Amyloid-*β* Measurements

Targeted CSF proteomics data were analyzed and processed by the Department of Neurology, Emory University School of Medicine, using CSF samples from the ADNI cohort by mass spectrometry. Detailed technical information on the mass spectrometry analysis of CSF, data acquisition, validation protocols, and quality control metrics used in this study are previously described. Briefly, the five protein targets (CHI3L1, NPTX2, SPP1, VGF, and YWHAZ) were previously identified by integrative proteomics as brain-based CSF biomarkers and reported to change in patients with Alzheimer's disease. CSF proteins were reduced, alkylated, denatured, and digested with Lys-C (1 : 100 enzyme to protein ratio) and trypsin (1 : 10 enzyme to protein ratio) and desalted. The resulting peptides were analyzed as a single replicate over approximately nine days using a standard flow Agilent 1290 Infinity II liquid chromatography system coupled with Thermo Fisher Scientific TSQ Altis Triple Quadrupole mass spectrometer. Isotopically labeled peptide standards were added for relative quantification by reporting the total area ratios for six targeted peptides related to five proteins (CHI3L1, NPTX2, SPP1, VGF, and YWHAZ).  Table 1 illustrates the peptides investigated, their associated genes, and their protein description.

In this study, all subjects were also tested for A*β*1-42, A*β*1-42/1-40 ratio, and A*β*1-42/1-38 ratio using tandem mass spectrometry analysis by the ADNI biomarker core at the University of Pennsylvania (UPenn). Detailed information regarding the 2D-UPLC-tandem mass spectrometry method used for these analyses has previously been described. This new method has been re-validated and compared with the original, reference method for A*β* and has fulfilled the requirements for validation as a rugged and reliable procedure. Each reported value in the data is the average of analyses of duplicate 0.1 mL aliquots from each CSF sample.

### 2.4. Statistical Analysis

The descriptive data were summarized using means and standard deviations for continuous variables and frequency and percentage for categorical variables. One-way analysis of variance (ANOVA) was applied for comparing demographics, cognitive assessments, and CSF-targeted proteomics and amyloid-*β* measurements between CN, MCI, and AD subjects. To determine the association between CSF proteomics and amyloid-*β* ratios and cognitive assessments, a multivariate linear regression model was used. In these models, CSF proteomics were entered as predictors, and age and gender were entered as covariates to control the effects of them as possible confounders. *R* square, standardized beta coefficient, and its *p* value were reported for these models. The data were analyzed using SPSS, Version 26, for Windows (SPSS Inc., Chicago, IL, USA). *p* values of less than 0.05 were considered statistically significant.

## 3. Results

A total of 719 subjects, including 225 CN, 381 MCI, and 113 AD subjects were investigated in this research. Significant differences in age, gender, education, and all cognitive assessments were observed between groups. A*β* and proteomics findings also showed significant differences between the three diagnosis groups.  Table 2 shows demographics, cognitive assessments, and A*β* and proteomics measurements.

### 3.1. Association between Proteomics and A*β*

Linear regression model analysis between A*β*1-42, A*β*1-42/1-40 ratio, and A*β*1-42/1-38 ratio and targeted proteomics controlled for age and gender as possible confounders in each CN, MCI, and AD group are shown in Table 3.

Among CN participants, A*β*1-42 showed a significant positive association with all six targeted proteomics; however, the A*β*1-42/1-40 ratio was significantly negatively associated with VVSSIEQK and GDSVVYGLR (*p* = 0.029 and 0.039, respectively), and A*β*1-42/1-38 was only associated negatively with GDSVVYGLR (*p* = 0.018). In the MCI group, although a significant association of A*β*1-42 with VAELEDEK and EPVAGDAVPGPK was observed (*p* < 0.001 for both), both A*β*1-42/1-40 and A*β*1-42/1-38 ratios showed negative significant association with other four proteomics. Among subjects with AD, IASNTQSR, VAELEDEK, and EPVAGDAVPGPK had a significant positive association with A*β*1-42 (*p* = 0.005, *p* < 0.001, and *p* < 0.001, respectively). However, a different pattern was observed for rations. VVSSIEQK, GDSVVYGLR, and QETLPSK showed significant association with both A*β*1-42/1-40 and A*β*1-42/1-38 ratios.

### 3.2. Association between Proteomics and Cognitive Assessments

Linear regression model analysis between cognitive measurements (CDR, ADAS-Cog 11 and 13, and MMSE) and targeted proteomics controlled for age and gender in CN, MCI, and AD group are shown in Table 4. In the CN group, only VAELEDEK and EPVAGDAVPGPK showed a significant negative association with both ADAS-Cog 11 and 13. No significant association was observed between any proteomics and CDR and MMSE scores. IASNTQSR, VAELEDEK, and VVSSIEQK were significantly associated with CDR, ADAS-Cog 11, and 13 in subjects with MCI; however, a different pattern was observed for MMSE which was significantly associated with VVSSIEQK, GDSVVYGLR, and QETLPSK. In AD patients, however, no significant association was yielded regarding CDR and MMSE scores, and VVSSIEQK was the only proteomic to associate with ADAS-Cog 11 and 13.

## 4. Discussion

In this study, we explored the correlation between CSF proteomics with A*β*1-42, A*β*1-42/1-40 ratio, and A*β*1-42/1-38 ratio with ADAS-total and ADAS-Cog-13. CSF proteomics has not been a trend of research in AD biomarker search so far [32]. One work suggested that the lower plasma A*β* 42/A*β* 40 was associated with greater cognitive decline in attention, executive function, and memory tests, besides the lower CSF A*β*42 level related to more and steeper cognitive in MMSE [33]. A*β*42 or A*β*42/A*β*40 in both plasma and CSF was also associated with delayed memory recall, the third item of ADAS-Cog, and they did not find any significant association between A*β* 42 or A*β*42/A*β*40 levels of plasma with delayed memory recall, but the CFS level of them was correlated with worse delayed memory recall (A*β*42, *β* = −0.124, *p* < 0.0001; A*β*42/A*β*40 ratio, *β* = −0.137, *p* < 0.0001) [34]. Moreover, studies showed that the decrease in CSF A*β*42 levels comes before AD for at least 10-20 years [35–37].

Our findings showed that A*β*1-42 was significantly reduced in cases with AD compared to both MCI and CN subjects. Similar findings were detected regarding the A*β* 1-42/1-40 and A*β* 1-42/1-38. Regarding the investigated peptides, IASNTQSR, VVSSIEQK, GDSVVYGLR, and QETLPSK indicated a significantly increased pattern in those with more progressive stages of the disease, while VAELEDEK and EPVAGDAVPGPK manifested a decreasing pattern in more severe cases. In the IASNTQSR peptide, which is related to the CHI3L1 protein gene, a significant correlation was detected with A*β* 1-42/1-40 at MCI and AD cases. This finding is in agreement with another study showing that CHI3L1 expression in the brain increases with aging, and its expression is higher in women than in men. This peptide is related to inflammatory processes and connected to neurodegeneration [38–40]. We found a positive correlation between the level of IASNTQSR and both ADAS-11 and ADAS-13 in MCI cases; however, no significant association was found between this peptide and cognitive scores at CN and AD subjects. This result suggests that the IASNTQSR has potential utility as a biomarker for the early stages of the disease [40]. Considering the VVSSIEQK peptide, Miller et al. showed that the expression of YWHAZ as a crucial linker gene decreases in both aging and AD [20]. Another study indicated that YWHAZ protein expression is reduced in the hippocampus of AD patients [41]; conversely, in the prefrontal lobe of mice, the expression level of the YWHAZ gene was increased. These may imply that the expression alteration can be different across brain regions [42]. We found a positive correlation between MMSE scores and QETLPSK in MCI cases. In addition, in this study, VVSSIEQK showed a correlation with CDR, ADAS-11, ADAS-13, and MMSE in those with MCI but no significant correlation was found for this peptide with cognitive scores at any other disease stage. As for the VALEDEK peptide, which is related to the NPTX2 protein, recent studies showed lower NPTX2 levels in individuals subjected to AD and a strong correlation with memory decline [4, 5]. One study found that NPTX2 plays an urgent role in Parkinson's disease [43, 44], and another study found that the level of CSF synaptic protein named NPTX2 considerably declines in AD patients. This protein can be known as a biomarker for cognitive decline. Currently, NPTX2 has been confirmed as a closely related protein to the pathogenesis of AD and can predict cognitive decline in mild AD and MCI [45]. One study looked for a correlation between serum level NPTX2 level and MOCA scores in patients with vascular dementia and find a positive correlation between them (*R* = 0.347, *p* = 0.042). These patients had a lower serum level (*p* < 0.001) of NPTX2 than the control group [46]. Our experience contradicts the previous findings of a moderate negative correlation between the peptide of this protein and ADAS-Total and ADAS-cog-13 in the A*β* + group and suggests that a lower level of VAELEDEK is correlated with more severe forms of the disease. With regard to the GDSVVYGLR and QETLPSK peptides, which are related to SPP1, one study showed a positive correlation between SSP1 and cognitive decline in MMSE scores in AD patients [19]. However, another study found a negative correlation between CSF-related SPP1 protein gene and MMSE scores in a control group, which shows a lower level of SSP1 and a higher level of MMSE scores and found a weak positive correlation between SSP1 and MMSE in MCI and AD patients [47]. Our results about those peptides associated with the SPP1 protein gene and cognitive decline in the ADAS indicated positive significant correlations for VVSSIEQK at the MCI phase represented in CDR, ADAS-11, and ADAS-13. Eventually, the QETLPSK peptide was only significantly correlated with MMSE in MCI subjects. Additionally, GDSVVYGLR showed a significant negative correlation with MMSE in MCI cases, which supports previous studies. Regarding the peptide EPVAGDAVPGPK, which is related to VGF, recent studies showed that VGF peptide levels in CSF are lower in patients with AD compared with controls [48, 49]. Although the mechanism behind the decrease in the level of VGF in CFS of AD patients is not clear yet [49], a lower level of the VGF protein gene can be influenced by a decrease in the level of NPTX2 [50]. One research examined the level of VGF-related peptides in CSF measured by ELISA among patients with dementia with Lewy bodies (DLB), patients with AD, and the control group. They found lower levels of CSF VGF in patients with DLB compared to AD and control groups, and in contrast with previous studies, VGF levels had not differed significantly between patients with AD and the control group [51]. Other previous studies showed that VGF peptide or protein levels were reduced in the CSF [16, 52, 53] and parietal cortex of a patient with AD [54] in comparison to the control group. All peptides related to VGF are decreased in AD patients [49]. In our study, however, no significant association was found between EPVAGDAVPGPK and cognitive score in MCI or AD cases, and significant associations were only detected in CN cases and ADAS-11 ADAS-13. This is a cross-sectional study, which limits the possibility of concluding the direction of associations. Furthermore, future studies are to consider the correlation between each item of ADAS with peptides and the A*β* ratio.

In conclusion, our results suggest the following peptides of IASNTQSR, VAELEDEK, VVSSIEQK, GDSVVYGLR, EPVAGDAVPGPK, and QETLPSK, correspond to formerly well-established biomarkers of A*β*. Particularly, CSF proteomics appears to be a considerable biomarker of cognitive decline in cases with MCI. Future longitudinal studies are required to investigate further the potential diagnostic and prognostic utilities of this proteomics longitudinally.

## Figures and Tables

**Table 1:** The investigated peptides, their associated genes, and the protein description.

Peptide	Protein gene	Protein description
IASNTQSR	CHI3L1	Chitinase-3-like protein 1
VAELEDEK	NPTX2	Neuronal pentraxin-2
QETLPSK	SPP1	Osteopontin
GDSVVYGLR	SPP1	Osteopontin
EPVAGDAVPGPK	VGF	Neurosecretory protein
VVSSIEQK	YWHAZ	14-3-3 protein zeta/delta

**Table 2:** Demographics and clinical characteristics of the study population.

	CN	MCI	AD	*p* value
Number	225	381	113	
Age (years)	73.06 ± 6.06	71.17 ± 7.58	74.27 ± 8.33	<0.001
Gender (F/M)	126/99	173/208	45/68	0.007
Education (years)	16.64 ± 2.51	16.24 ± 2.62	15.74 ± 2.69	0.011
Cognitive assessments				
CDR	0.05 ± 0.16	1.42 ± 0.85	4.60 ± 1.71	<0.001
ADAS-11	5.76 ± 2.98	9.17 ± 4.51	20.43 ± 7.25	<0.001
ADAS-13	8.92 ± 4.40	14.75 ± 6.90	30.27 ± 9.21	<0.001
MMSE	29.08 ± 1.15	28.07 ± 1.73	23.21 ± 1.98	<0.001
Amyloid *β*				
A*β*1-42	1449.60 ± 646.48	1180.90 ± 581.23	811.75 ± 458.56	<0.001
A*β*1-42/1-40	0.17 ± 0.05	0.14 ± 0.06	0.10 ± 0.03	<0.001
A*β*1-42/1-38	0.73 ± 0.24	0.62 ± 0.25	0.45 ± 0.17	<0.001
Targeted proteomics				
IASNTQSR	0.3987 ± 0.1389	0.4056 ± 0.1392	0.4780 ± 0.1656	<0.001
VAELEDEK	0.0039 ± 0.0013	0.0037 ± 0.0012	0.0031 ± 0.0011	<0.001
VVSSIEQK	0.0230 ± 0.0090	0.0261 ± 0.0115	0.0330 ± 0.0110	<0.001
GDSVVYGLR	0.3613 ± 0.1286	0.3785 ± 0.1439	0.4222 ± 0.1493	0.001
EPVAGDAVPGPK	0.1390 ± 0.0662	0.1274 ± 0.0574	0.1148 ± 0.0817	0.004
QETLPSK	1.3751 ± 0.4928	1.4631 ± 0.5832	1.6330 ± 0.5515	<0.001

CN: cognitively normal; MCI: mild cognitive impairment; AD: Alzheimer's disease.

**Table 3:** Linear regression between amyloid-*β* ratios and targeted proteomics.

	CN			MCI			AD		
	*R* square	Beta coefficient	*p* value	*R* square	Beta coefficient	*p* value	*R* square	Beta coefficient	*p* value
*Aβ1-42*									
IASNTQSR	0.089	0.298	**<0.001**	0.054	0.089	0.095	0.084	0.291	**0.005**
VAELEDEK	0.262	0.501	**<0.001**	0.138	0.315	**<0.001**	0.133	0.347	**<0.001**
VVSSIEQK	0.049	0.193	**0.006**	0.047	-0.020	0.708	0.020	0.081	0.401
GDSVVYGLR	0.044	0.171	**0.011**	0.050	-0.054	0.287	0.017	0.057	0.549
EPVAGDAVPGPK	0.328	0.564	**<0.001**	0.179	0.371	**<0.001**	0.238	0.481	**<0.001**
QETLPSK	0.068	0.233	**<0.001**	0.048	-0.033	0.512	0.021	0.092	0.344
*Aβ1-42/1-40*									
IASNTQSR	0.100	-0.064	0.361	0.111	-0.245	**<0.001**	0.087	-0.203	**0.046**
VAELEDEK	0.100	0.063	0.330	0.058	-0.031	0.558	0.060	-0.084	0.370
VVSSIEQK	0.116	-0.146	**0.029**	0.134	-0.287	**<0.001**	0.142	-0.303	**0.001**
GDSVVYGLR	0.114	-0.132	**0.039**	0.146	-0.300	**<0.001**	0.132	-0.281	**0.002**
EPVAGDAVPGPK	0.102	0.075	0.244	0.062	-0.066	0.196	0.053	-0.008	0.934
QETLPSK	0.101	-0.070	0.278	0.125	-0.262	**<0.001**	0.110	-0.242	**0.010**
*Aβ1-42/1-38*									
IASNTQSR	0.092	-0.064	0.360	0.107	-0.255	**<0.001**	0.059	-0.162	0.115
VAELEDEK	0.090	0.035	0.586	0.051	-0.048	0.355	0.046	-0.097	0.303
VVSSIEQK	0.100	-0.112	0.097	0.131	-0.299	**<0.001**	0.112	-0.278	**0.003**
GDSVVYGLR	0.112	-0.153	**0.018**	0.141	-0.308	**<0.001**	0.100	-0.250	**0.007**
EPVAGDAVPGPK	0.092	0.051	0.435	0.057	-0.094	0.066	0.039	-0.048	0.618
QETLPSK	0.096	-0.084	0.197	0.115	-0.261	**<0.001**	0.078	-0.206	**0.029**

CN: cognitively normal; MCI: mild cognitive impairment; AD: Alzheimer's disease. *p* values < 0.05 are in bold.

**Table 4:** Linear regression between cognitive assessments and targeted proteomics.

	CN			MCI			AD		
	*R* square	Beta coefficient	*p* value	*R* square	Beta coefficient	*p* value	*R* square	Beta coefficient	*p* value
*CDR*									
IASNTQSR	0.014	0.095	0.197	0.029	0.170	**0.002**	0.004	0.013	0.898
VAELEDEK	0.007	-0.033	0.629	0.017	-0.122	**0.023**	0.013	-0.095	0.321
VVSSIEQK	0.006	0.012	0.867	0.019	0.129	**0.016**	0.024	0.142	0.141
GDSVVYGLR	0.013	0.087	0.199	0.007	0.057	0.274	0.006	0.042	0.660
EPVAGDAVPGPK	0.008	0.046	0.495	0.006	-0.051	0.333	0.011	-0.084	0.385
QETLPSK	0.015	0.093	0.168	0.008	0.068	0.190	0.006	-0.041	0.676
*ADAS-11*									
IASNTQSR	0.145	-0.091	0.183	0.093	0.142	**0.007**	0.017	0.115	0.271
VAELEDEK	0.160	-0.148	**0.018**	0.097	-0.156	**0.002**	0.013	-0.084	0.383
VVSSIEQK	0.146	-0.093	0.157	0.106	0.184	**<0.001**	0.090	0.294	**0.002**
GDSVVYGLR	0.145	-0.085	0.179	0.076	0.035	0.481	0.013	0.084	0.379
EPVAGDAVPGPK	0.162	-0.154	**0.014**	0.083	-0.094	0.064	0.014	-0.092	0.345
QETLPSK	0.143	-0.066	0.296	0.079	0.069	0.165	0.009	0.058	0.549
*ADAS-13*									
IASNTQSR	0.177	-0.082	0.221	0.129	0.176	**0.001**	0.019	0.145	0.166
VAELEDEK	0.195	-0.156	**0.011**	0.126	-0.165	**0.001**	0.004	-0.055	0.564
VVSSIEQK	0.172	-0.037	0.565	0.149	0.238	**<0.001**	0.076	0.278	**0.004**
GDSVVYGLR	0.175	-0.064	0.298	0.106	0.069	0.161	0.008	0.085	0.375
EPVAGDAVPGPK	0.192	-0.146	**0.017**	0.108	-0.086	0.083	0.005	-0.066	0.496
QETLPSK	0.174	-0.057	0.360	0.109	0.092	0.060	0.006	0.070	0.472
*MMSE*									
IASNTQSR	0.034	-0.141	0.053	0.065	-0.102	0.053	0.008	0.000	0.998
VAELEDEK	0.018	-0.021	0.757	0.059	0.063	0.226	0.023	0.122	0.202
VVSSIEQK	0.018	-0.017	0.807	0.078	-0.157	**0.002**	0.023	-0.126	0.192
GDSVVYGLR	0.026	0.093	0.165	0.066	-0.102	**0.043**	0.010	0.049	0.606
EPVAGDAVPGPK	0.017	0.005	0.946	0.055	-0.012	0.817	0.013	0.072	0.459
QETLPSK	0.022	0.065	0.333	0.071	-0.127	**0.012**	0.010	0.042	0.662

CN: cognitively normal; MCI: mild cognitive impairment; AD: Alzheimer's disease. *p* values < 0.05 are in bold.

## Data Availability

The data used in this research was obtained from Alzheimer's Disease Neuroimaging Initiative (ADNI) and is available with permission to all researchers.
